# Private Medical Instruction

**Published:** 1846-09

**Authors:** 


*Private Medical Instruction.*—We have received, within a few days, the circulars of two private schools, one, at Philadelphia, under the management of James M'Clintock, M. D., and the other lately instituted by Drs. Van Buren and Isaacs, of New-York city, under the patronage of the University of the city of New-York.

Dr. M'Clintock has the reputation of being an accomplished anatomist and surgeon, and of possessing, in an eminent degree, the faculty of imparting knowledge to the student. It is sufficient evidence of the favor with which his labors are regarded, that a class of 165 attended his courses during the winter of 1845–6. We see by his announcement that he will continue his instructions the coming winter, embracing dissections, lectures on anatomy, physiology, surgical anatomy, and operative surgery, together with examinations on all the branches taught in medical institutions. He is assisted in his duties by Drs. White, McClosky and Van Stavoren. We do not doubt that medical students who design to pass the winter in Philadelphia, will find it for their advantage to avail themselves of the facilities and assistance which this school offers. We notice in the catalogue the names of several practitioners, and, also, medical officers of the Army and Navy.

The labors of Drs. Van Buren and Isaacs commence with the following winter. In the character and competency of these gentlemen, however, the student who selects the city of New-York as the theatre of his studies, may have every assurance that his expectations will not be disappointed.

We regard private medical instruction as a most important auxiliary to didactic teaching by means of formal lectures; and if we were to single out any one defect in the present system of medical education which, in our estimation, is productive of more evil than any other, it would be the fact that aside from the courses of lectures generally prescribed by law as requisites for graduation, a large proportion of medical pupils receive little or no instruction. They enter a physician's office, and are allowed the privilege of reading as their fancy, or as chance may direct, the few books which constitute his library. The physician rarely takes pains to guide their studies, and provide late works and modern improvements for their advantage; how should the latter be expected, when he does not deem them necessary for his own benefit? Still less does he pursue any regular system of examinations, and oral teaching. Is it strange, that under such circumstances, little progress is made, and that not unfrequently the errors imbibed, which are afterward to be unlearned, preponderate over the actual knowledge which is acquired! Too often the relation which subsists between the practitioner and his office students is so far from that denoted by the the terms *teacher* and *pupil*, that it is a mere sham; and if the fact were not obscured by custom, it could not fail to appear, as it really is, an imposition on the pupil, the profession and the public. This seems to us a serious evil, and we would respectfully commend it to the attention of those who think that medical schools are responsible for all the ignorance and unskilfulness which are to be found in the ranks of the profession. Can any doubt that a reform is here needed? that to enter one's name as a medical pupil should mean something more than to "*keep office*," and make onesself generally useful; and that the position of an instructor should involve the responsibilities and duties of communicating instruction, and exercising supervision over the studies of the novitiate? Three years of medical pupilage is certainly not too long, admitting that every portion of the period is devoted actually to study, with the aid of constant instruction; and there can be no doubt that the average of professional acquirement would be not a little raised, if the situation of the majority of students in the intervals between the lecture terms were more favorable for improvement. With these views, we are glad to see private medical schools established in different parts of the country by those who are led by their tastes and talents to engage in the vocation of teachers, and the fact that such enterprises are appreciated and sustained, affords good evidence that the interests of medical education are in the ascendant.

